# Capacity of astrocytes to promote axon growth in the injured mammalian central nervous system

**DOI:** 10.3389/fnins.2022.955598

**Published:** 2022-09-20

**Authors:** Matin Hemati-Gourabi, Tuoxin Cao, Megan K. Romprey, Meifan Chen

**Affiliations:** ^1^Spinal Cord and Brain Injury Research Center, Lexington, KY, United States; ^2^Department of Neuroscience, University of Kentucky, Lexington, KY, United States

**Keywords:** reactive astrocytes, axon regeneration, axon sprouting, CNS injury, glial scar, spinal cord injury, neural repair, astrogliosis

## Abstract

Understanding the regulation of axon growth after injury to the adult central nervous system (CNS) is crucial to improve neural repair. Following acute focal CNS injury, astrocytes are one cellular component of the scar tissue at the primary lesion that is traditionally associated with inhibition of axon regeneration. Advances in genetic models and experimental approaches have broadened knowledge of the capacity of astrocytes to facilitate injury-induced axon growth. This review summarizes findings that support a positive role of astrocytes in axon regeneration and axon sprouting in the mature mammalian CNS, along with potential underlying mechanisms. It is important to recognize that astrocytic functions, including modulation of axon growth, are context-dependent. Evidence suggests that the local injury environment, neuron-intrinsic regenerative potential, and astrocytes’ reactive states determine the astrocytic capacity to support axon growth. An integrated understanding of these factors will optimize therapeutic potential of astrocyte-targeted strategies for neural repair.

## Introduction

Failure of axons to regenerate in the adult mammalian CNS results in persisting functional deficits following CNS insults (Chen and Zheng, 2014). Overcoming barriers to axon regrowth is critical to restoration of neural functions. Besides the limited growth potential of mature CNS neurons, a major impediment to axon growth is the scar tissue—or widely referred to as the glial scar—at the site of acute focal damage. Regeneration-inhibitory activity of the glial scar has been excellently reviewed (Silver and Miller, 2004; Silver et al., 2014; Bradbury and Burnside, 2019). While astrocytes at the lesion border have been widely reputed to be a barrier to axon growth by association with the multicellular scar tissue, cell type-specific interrogation of gene expression and functions suggest that astrocytes are not principally responsible for regenerative failure at the lesion. Accumulating evidence suggests that the capacity of astrocytes to support axon growth in an injured CNS, at and remote from the lesion, may be greater than previously appreciated. In forming an understanding of astrocyte-mediated effects on axon growth, it is important to recognize that they are contextual—influenced at least in part by the local injury environment, neuron-intrinsic regenerative capacity, and astrocytes’ reactive states. This review will compare the astrocytic response to CNS injury between humans and mice, discuss evidence for a positive role of astrocytes in supporting axon regeneration at the lesion and axon sprouting away from the lesion, present potential underlying mechanisms, discuss the diversity of astrocytic injury responses with respect to their innate heterogeneity, and consider the role of the local injury environment in determining astrocytes’ capacity to facilitate axon growth, with the goal to broaden understanding of astrocytes’ potential for neural repair.

## Astrocytic response to central nervous system injury in humans and mice

Astrocytes react to CNS injury with a range of phenotypic and functional changes that is broadly referred to as reactive astrogliosis (Sofroniew, 2014; Escartin et al., 2021). Following acute focal injury to the CNS (traumatic and ischemic injuries to the brain and spinal cord), scar-forming astrogliosis occurs at the lesion site to isolate tissue damage, where astrocytes upregulate glial fibrillary acidic protein (GFAP), hypertrophy, proliferate, and overlap their cellular processes to form a dense astrocytic border that encloses a lesion core of non-neural cells (Burda and Sofroniew, 2014; Silver et al., 2014). It is important to distinguish the astrocytic scar from the glial scar (Escartin et al., 2021). Although these two terms have been historically synonymous, use of “glial scar” has evolved to broadly describe the entire scar tissue that is a multicellular structure with a GFAP^+^ astrocytic lesion border and a GFAP^–^ lesion core (Wanner et al., 2013; Zhu et al., 2015; Chen et al., 2018). Therefore, to avoid confusion, and as the field continues to gain cell type-specific understanding of the origin and function of scar components, it would be most informative to specify the cell types rather than using the umbrella term of “glial scar” in discussion of the scar tissue. The astrocytic scar, in this review, specifically refers to the astrocytic component that lines the lesion border. With increasing distance from the lesion, the level of astrocyte reactivity decreases and gradually transitions from proliferating and overlapping scar-forming astrocytes at the primary lesion, to hypertrophic stellate astrocytes that retain their tiling property in reactive tissue, then to non-reactive astrocytes as found in healthy tissue (Wanner et al., 2013). This is generally true for astrogliosis emanating from acute focal lesion in the gray matter. However, damage to white matter tracts or to neurons from which they originate results in non-scar forming astrogliosis in areas of Wallerian degeneration that can extend over a long distance without tapering off in astrocyte reactivity (Wang et al., 2009; Chen et al., 2022).

Astrocytes respond rapidly to blood-brain barrier (BBB) damage. It is postulated that BBB damage at the lesion site creates a gradient of blood-borne immune cells and damage associated molecules that elicits a tapering astrocytic response in the injured spinal cord (Burda and Sofroniew, 2014). Gradation of astrogliosis is conserved between humans and mice (Sofroniew and Vinters, 2010), with some differences in timing and severity of astrogliosis that is likely due to the lack of direct comparison in the type and phase of injury examined. Within days after spinal cord injury (SCI) in humans, or 1–2 days after injury in mice, reactive astrocytes display nuclear enlargement, elongated cellular processes, and cytoskeletal hypertrophy (Buss et al., 2004; Norenberg et al., 2004; Burda and Sofroniew, 2014). Astrocytic encapsulation of the injury site is observed weeks after injury in humans, or 2–3 weeks after injury in mice (Herrmann et al., 2008; Wanner et al., 2013; Burda and Sofroniew, 2014; Chen et al., 2018). In both humans and mice, the astrocytic scar persists chronically once established, present even 30 years after injury in humans (Buss et al., 2007).

Studies of reactive astrocytes in the context of acute focal CNS injury have primarily focused on scar-forming astrocytes at the lesion. Scar-forming astrocytes originate from *in situ* proliferation of adult astrocytes (Bush et al., 1999; Faulkner et al., 2004; Okada et al., 2006; Barnabe-Heider et al., 2010; Wanner et al., 2013), differentiation of NG2^+^ oligodendrocyte progenitor cells (Dimou et al., 2008; Barnabe-Heider et al., 2010; Komitova et al., 2011; Hackett et al., 2016, 2018), and minimally, differentiation from ependymal cells (Meletis et al., 2008; Barnabe-Heider et al., 2010; Sabelstrom et al., 2013; Zukor et al., 2013; Ren et al., 2017). Molecular regulators of scar-forming astrogliosis have been comprehensively reviewed (Bradbury and Burnside, 2019; Sofroniew, 2020). The astrocytic scar is neuroprotective in the early phase of acute focal injury, and essential to tissue integrity in both acute and chronic phases of traumatic injury (Faulkner et al., 2004; Okada et al., 2006; Herrmann et al., 2008; Anderson et al., 2016). Disruption of astrocytic scar formation, by genetic ablation of proliferating astrocytes or attenuation of scar formation, results in enlarged lesion volume, widespread inflammatory cell infiltration, extensive neural tissue degeneration, and exacerbated functional outcome (Pekny et al., 1999; Faulkner et al., 2004; Brambilla et al., 2005; Okada et al., 2006; Herrmann et al., 2008; Sahni et al., 2010; Chen et al., 2018). Historically synonymous with the glial scar, the astrocytic component has been reputed as physical and chemical barriers to axon growth in the chronic phase of injury (Silver and Miller, 2004; Bradbury and Burnside, 2019). The extent of scar-forming astrogliosis in human SCI varies from “an impenetrable barrier is practically never seen” (Norenberg et al., 2004) to “dense GFAP-positive matrix can be seen after long survival times” (Buss et al., 2004). This divergence of observations seems to stem from difference in injury severity, with the formation of a dense astrocytic scar in human cases defined as “complete lesions without remaining nerve fibers traversing the lesion center” (Buss et al., 2004). This raises an intriguing possibility that the astrocytic scar may not be as much of a physical impediment to cellular regeneration in human survivors with anatomically incomplete SCI. The chemical barrier presented by the astrocytic scar has been mainly attributed to the production of chondroitin sulfate proteoglycans (CSPGs), which are extracellular matrix molecules generally regarded as inhibitory to axon growth (Davies et al., 1999; Silver and Miller, 2004). As methodological advances continue to improve cellular and molecular resolution of scar tissue composition, it is now known that scar-forming astrocytes are not the sole producers of CSPGs (Silver et al., 2014; Anderson et al., 2016). Supporting this are recent findings that oligodendrocyte progenitor cells (OPCs) at the lesion abundantly and preferentially express axon growth inhibitory CSPGs compared to astrocytes, as revealed by single-cell RNA sequencing (Milich et al., 2021; Wahane et al., 2021). Furthermore, scar-forming astrocytes produce axon growth-permissive extracellular matrix (ECM) molecules (Liesi et al., 1984; Frisen et al., 1995; Anderson et al., 2016), suggesting a potential to support axon growth (discussed in section “Evidence for astrocytes supporting axon regeneration”). As the levels of CSPGs within the scar tissue change temporally following injury, the relative abundance and interaction of growth inhibitory vs. permissive ECM molecules within the scar tissue likely contribute to determine the permissive window for axon regeneration at the lesion (McKeon et al., 1995).

In contrast, the role of moderately reactive astrocytes, which have also been referred to as stellate or diffuse reactive astrocytes, in neural repair remains elusive. This type of reactive astrocytes so far has only been recognized as morphologically distinct from scar-forming astrocytes (Anderson et al., 2014). Remote from the lesion site that is hostile to axon growth, stellate reactive astrocytes may have higher capacity to facilitate axon growth (see section “Evidence for astrocytes supporting axon sprouting”). In a mouse model that genetically stimulates stellate reactive astrocytes, their ability to promote axon sprouting distal to the primary lesion was revealed (see section “Evidence for astrocytes supporting axon sprouting”).

## Evidence for astrocytes supporting axon regeneration

Axon regeneration is defined here as the regrowth of injured axons. In the mature mammalian CNS, damaged axons cannot spontaneously regenerate. The supportive role of astrocytes in adult CNS axon regeneration was revealed under experimental conditions that enhanced axons’ intrinsic capacity to grow. Phosphatase and tensin homolog (*Pten)* gene deletion or knockdown in corticospinal neurons promotes spontaneous regeneration of severed corticospinal axons after spinal cord injury (Liu et al., 2010; Zukor et al., 2013; Du et al., 2015). Under these conditions, while only a small subset of corticospinal axons regenerated into the primary lesion, a majority of these *Pten*-deleted regenerating axons grew along thin GFAP^+^ tissue bridges that developed across the lesion epicenter (Liu et al., 2010; Zukor et al., 2013; Du et al., 2015). Association of the GFAP^+^ matrix with regenerating axons has been consistently observed following dorsal hemisection, complete crush, or complete transection of the spinal cord at thoracic level T8 in adult mice (Lee et al., 2010; Liu et al., 2010; Zukor et al., 2013; Du et al., 2015). In contrast, regenerating axons did not contact the GFAP^–^ lesion core (Lee et al., 2010; Liu et al., 2010; Zukor et al., 2013). These findings together suggest that the GFAP^+^ matrix can be axon growth-permissive, a property that may belong to a subtype of scar-forming astrocytes, or may be induced by axons stimulated to grow. It was reported that “although only 42% of the lesion is GFAP^+^, 80% of the axons in the lesion are in the GFAP^+^ portion” (Zukor et al., 2013). Notably, extension of GFAP^+^ bridge and accompanying axon regeneration into the lesion were observed in the chronic phase of injury (Lee et al., 2010; Liu et al., 2010; Zukor et al., 2013; Du et al., 2015), following initial establishment of the astrocytic scar with a well-demarcated GFAP^+^ and GFAP^–^ lesion boundary in the subacute phase of injury (Okada et al., 2006; Herrmann et al., 2008; Wanner et al., 2013; Zhu et al., 2015; Hara et al., 2017; Chen et al., 2018). These observations suggest injury phase-dependent regulation of astrocytic functions: isolation of tissue damage in the early phase and potential facilitation of axon growth at a later stage.

Lineage tracing showed that GFAP^+^ bridge-forming cells are likely derived from resident mature astrocytes, with minimal contribution from progeny of ependymal cells in the adult spinal cord (Zukor et al., 2013). Axons regenerated along astroglial bridges 8–12 weeks after injury, but not earlier (Liu et al., 2010; Zukor et al., 2013). In fact, even when neuronal PTEN was deleted 1 year after injury, association of regenerating axons with astroglial bridges was still observed at 19 months after injury (Du et al., 2015), when the astrocytic scar had been chronically established. These findings show that in an environment conducive to the formation of astroglial bridges, their axon growth-supportive potential is unaffected by tissue maturation at the lesion. This contrasts the expectation that a more mature, thus more dense, astrocytic scar would be more obstructive to axon growth. It remains possible, however, that given the long survival period of this study, a more mature astrocytic scar may have diminished capacity to promote axon growth. Elucidating injury stage-specific permissibility of astrocytes to axon growth would be of interest for future investigation. Small lesions seem to favor astroglial bridge formation (Liu et al., 2010; Zukor et al., 2013; Du et al., 2015), as this process likely involves astroglial migration into the lesion from either lesion edge (White et al., 2008; Liu et al., 2010; Du et al., 2015). In the 129X1/SvJ strain of mice, substantial astrocyte migration into the lesion core is associated with robust regeneration of serotonergic and sensory axons into the lesion after midthoracic spinal cord contusion (Ma et al., 2004). Axon regrowth along astroglial bridges under experimental stimulation of neuronal regenerative potential also raises the possibility of bidirectional interaction between regenerating axons and astrocytes at the lesion, such that the growing axon may in turn induce a growth supportive phenotype of astrocytes (Silver, 2016).

Are astrocytes at the lesion required for axon regeneration? This was tested on sensory axons stimulated to regrow, *via* a peripheral conditioning lesion and provision of neurotrophic factors at the injury site, in a spinal cord injury model of complete crush at T10 (Anderson et al., 2016). Astrocytic scar formation was attenuated either by genetic ablation of proliferating astrocytes or gene deletion of signal transducer and activator of transcription (*STAT3*), a key regulator of astrocyte proliferation and reactivity (Herrmann et al., 2008; Wanner et al., 2013). Either approach of preventing astrocytic scar formation significantly reduced sensory axon regeneration, even when the neuron-intrinsic growth program was robustly stimulated (Anderson et al., 2016). These results indicate a positive role of reactive astrocytes on axon regeneration at the lesion. It should be taken into consideration, however, that testing the effects of scar-forming astrocytes on axon regeneration by a loss-of-function approach is inherently challenging because the astrocytic scar is required for tissue integrity in both the acute and chronic phases of injury (Bush et al., 1999; Faulkner et al., 2004), making it difficult to distinguish its direct effects on axon regeneration *per se* from indirect effects *via* tissue protection. Whether the astrocytic scar examined in this study had matured to a clinically relevant density, and how scar-forming astrocytes can form a cellular barrier to non-neural cells without restraining axon growth are key questions that have been raised (Silver, 2016). One possible answer to the latter question is that the activities of scar-forming astrocytes in corralling inflammatory cells and promoting axon growth are temporally separate over the course of injury. Alternatively, the molecular interface between spared tissue and astrocytes may be axon growth-permissive, whereas that between astrocytes and the non-neural lesion core may be growth inhibitory. Nevertheless, gene expression analysis revealed that scar-forming astrocytes upregulate extracellular matrix molecules that support axon growth, including laminins and beneficial subtypes of CSPGs (Anderson et al., 2016), in addition to growth-inhibitory CSPGs (Davies et al., 1999; Silver et al., 2014; Anderson et al., 2016) and type I collagen (Hara et al., 2017). Converging evidence of injury-dependent production of pro-regenerative molecules by reactive astrocytes at the lesion suggests their potential to support axon growth (Liesi et al., 1984; Frisen et al., 1995; Goss et al., 1998; Lee et al., 1998; Anderson et al., 2016).

Consistent with these findings, stimulation of astrocyte migration at the lesion site by TGFα administration enhanced axon growth into the lesion core following spinal cord contusion at T9 (White et al., 2008, 2011). Axons within the lesion were found to associate with astrocytes that expressed high levels of both growth-supportive laminin and the growth-inhibitory CSPG neurocan (White et al., 2008). Transplantation of astrocytes derived *in vitro* from rat glial-restricted precursors (GRPs) into cervical spinal cord lesion resulted in robust regeneration of sensory and rubrospinal axons at the lesion, concomitant with improved locomotor recovery following spinal cord injury (Davies et al., 2006). Similarly, transplantation of human GRPs, which differentiated into astrocytes *in vivo* following transplantation into cervical spinal cord lesion, stimulated regeneration and sprouting of rostral ventral respiratory group axons and improved diaphragm activity (Goulao et al., 2019). Immature rat cortical astrocytes, when co-transplanted with chondroitinase ABC that digests CSPGs, were also able to promote regeneration of basal forebrain axons following microlesion of the cingulum (Filous et al., 2010). These findings show that astrocytes, when stimulated to invade into or placed at the lesion site, have the capacity to facilitate axon regeneration.

## Evidence for astrocytes supporting axon sprouting

Following injury to the adult mammalian CNS, damaged axons fail to regenerate, but spared intact axons can spontaneously grow or sprout (Chen and Zheng, 2014). Injury-induced axon sprouting is a compensatory response that can occur away from the injury site to establish short local or relay connections with denervated neuronal targets (Chen and Zheng, 2014). Importantly, axon sprouting is a form of neural plasticity that contributes to neural recovery in human and animal models of CNS injury (Cafferty and Strittmatter, 2006; Fawcett et al., 2007; Ueno et al., 2012; Chen and Zheng, 2014; Wahl et al., 2014; Jin et al., 2015; Carmichael et al., 2017; Courtine and Sofroniew, 2019).

Intriguingly, astrocytes have been implicated to promote axon sprouting that occurs far from the primary injury site. Following unilateral photothrombotic stroke targeted to the forelimb sensorimotor cortex of one cerebral hemisphere, corticospinal axons that originate from the uninjured hemisphere sprout across the midline in the cervical spinal cord to project into the denervated side (Lindau et al., 2014; Liu et al., 2014; Wahl et al., 2014). Effects of reactive astrocytes on axon sprouting in this model were tested using mice with constitutive whole-body gene deletions of two structural proteins that are highly upregulated in reactive astrocytes: GFAP and vimentin (GFAP^–/–^Vim^–/–^) (Liu et al., 2014). GFAP^–/–^Vim^–/–^ mice exhibit impaired astrocytic reactivity, including attenuated cytoskeletal hypertrophy and enlarged lesion size in response to injury (Pekny et al., 1999; Wilhelmsson et al., 2004; Li et al., 2008). Following unilateral cortical photothrombosis, intra-spinal sprouting of corticospinal axons in GFAP^–/–^Vim^–/–^ mice was reduced, accompanied by impaired motor recovery (Liu et al., 2014). While contribution from other cell types cannot be ruled out due to global deletion of GFAP and vimentin, these findings suggest that astrocytes support axon sprouting. Interestingly, using the same GFAP^–/–^Vim^–/–^ mice, but a different CNS injury model of spinal cord injury at low thoracic level T12, sprouting of serotonergic and corticospinal axons was found to decrease at the lumbar spinal cord (Menet et al., 2003). The opposite sprouting response observed may be attributed to difference in injury type, as astrocytes reacting to ischemic stroke have been shown to display molecular signatures beneficial to repair (Zamanian et al., 2012).

The above studies raise the questions of what kind of astrocytes regulate axon sprouting and how. Given that axon sprouting examined occurs distal to the lesion, it is conceivable that non-scar forming reactive astrocytes located away from the lesion play a role. In line with this, reactive astrocytes before scar formation (Hara et al., 2017) or located in spared tissue away from the injury site (Davies et al., 1999) have been shown to possess greater potential to support axon growth than scar-forming astrocytes at the lesion (Davies et al., 1999; Hara et al., 2017). If moderately reactive astrocytes are indeed more capable of supporting axon growth, can this type of reactive astrocytes be amplified to promote axon plasticity after CNS injury?

A mouse model was developed to modulate astrocyte reactivity through gene expression manipulation of leucine zipper-bearing kinase (LZK) in astrocytes, which was identified as an activator of STAT3 signaling (Chen et al., 2018). In the absence of CNS injury, LZK overexpression in adult astrocytes activates STAT3 signaling and stimulates moderate astrocyte reactivity throughout the CNS gray matter, as assessed by the hallmarks of astrogliosis (including upregulation of GFAP, vimentin, cytoskeletal hypertrophy, cell proliferation, and STAT3 activation), while preserving individual astrocyte domains (Chen et al., 2018). These characteristics render them similar to stellate reactive astrocytes, defined as hypertrophic GFAP^+^ reactive astrocytes that can proliferate, but maintain their tiling property and stellate appearance that resembles mature astrocytes in healthy tissue (Wanner et al., 2013; Anderson et al., 2014). Genetic stimulation of stellate reactive astrocytes *via* LZK induction in adult astrocytes enhanced corticospinal axon sprouting in the spinal cord following unilateral photothrombotic stroke of the primary motor cortex (Chen et al., 2022). Furthermore, this phenomenon is injury-dependent (Chen et al., 2022). LZK-stimulated stellate reactive astrocytes also produce the cytokine ciliary neurotrophic factor (CNTF) (Chen et al., 2022), which is known to be a sufficient and potent promoter of CNS axon sprouting (Jin et al., 2015). While it remains to be determined the extent to which CNTF accounts for the axon sprouting stimulatory effects of LZK and how these stellate reactive astrocytes impact neuronal functions, these findings nevertheless uncovered an exciting positive role of stellate astrogliosis in axon sprouting, with important implications for shaping neural plasticity in an injured CNS. As further discussed in section “Astrocyte diversity: an astrocyte subtype that promotes axon growth?,” astrocytes’ capacity to support axon regeneration vs. axon sprouting may differ depending on the local injury environment, neuron-intrinsic regenerative potential, and innate heterogeneity of astrocytes.

## Astrocyte-based mechanisms of promoting axon growth

**FIGURE 1:**
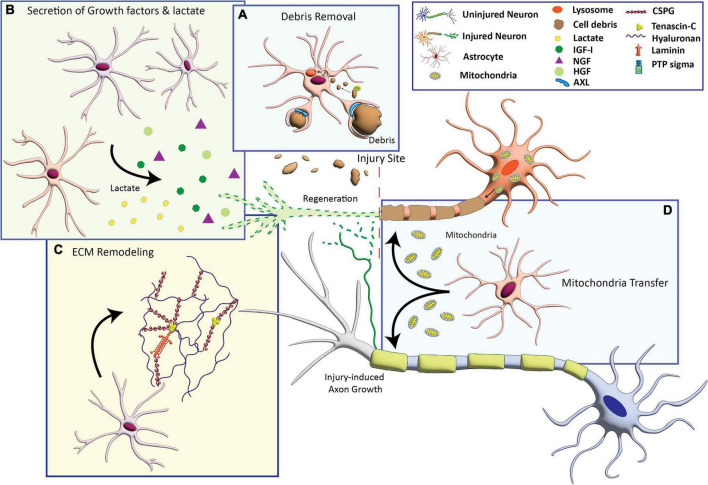
Astrocyte-based mechanisms that can promote axon growth. **(A)** Activation of phagocytosis to engulf cellular debris that inhibit axon growth. **(B)** Secretion of growth factors including insulin-like growth factor (IGF-I), nerve growth factor (NGF), and hepatocyte growth factor (HFG), and the metabolite lactate. **(C)** Production of extracellular matrix (ECM) molecules including laminin and axon growth-permissive chondroitin sulfate proteoglycans (CSPGs). **(D)** Transfer of healthy mitochondria to compromised neurons to provide bioenergetic support for axon growth.

Potential mechanisms that underlie axon growth-supportive effects of astrocytes include the production of neurotrophic factors, remodeling of the extracellular matrix, clearance of myelin debris, and provision of bioenergetic support for axon growth, discussed below (Figure 1).

### Neurotrophic factor production

Reactive astrocytes produce a number of neurotrophic factors upon spinal cord and brain injuries. These include brain-derived neurotrophic factor (BDNF) known to promote neuronal survival and axon growth (Dougherty et al., 2000; Ikeda et al., 2001); ciliary neurotrophic factor (CNTF) (Lee et al., 1998; Tripathi and McTigue, 2008; Chen et al., 2022) shown to robustly stimulate axon regeneration and sprouting of CNS axons (Muller et al., 2007; Jin et al., 2015); nerve growth factor (NGF) (Goss et al., 1998; Krenz and Weaver, 2000) that enhances axon regrowth (Tuszynski et al., 2002; Hannila and Kawaja, 2005; Mesentier-Louro et al., 2019) using reactive astrocytes as a substrate (Kawaja and Gage, 1991); fibroblast growth factor 2 (FGF2) (do Carmo Cunha et al., 2007) shown to promote CNS axon branching (Szebenyi et al., 2001) and sensory axon regeneration (Lee et al., 2017); hepatocyte growth factor (HGF) (Shimamura et al., 2007) capable of increasing CNS axon regrowth (Kitamura et al., 2007; Yamane et al., 2018); and insulin-like growth factor 1 (IGF1) (Yao et al., 1995) with potent regeneration-stimulatory activity on corticospinal axons (Hollis et al., 2009; Liu et al., 2017). Human spinal cord astrocytes, when stimulated with IL1β to undergo astrogliosis *in vitro*, also express the growth factors such as FGF2, BDNF, and NGF, adapting an overall axon growth-permissive phenotype (Teh et al., 2017).

### Extracellular matrix remodeling

Scar-forming reactive astrocytes are one of numerous cell types at the lesion that produce CSPGs (Morgenstern et al., 2002; Silver et al., 2014; Anderson et al., 2016; Milich et al., 2021; Wahane et al., 2021), which may be more inhibitory to axon regeneration than degenerating CNS myelin (Davies et al., 1999). It is proposed that CSPGs interfere with axon growth by stabilizing dystrophic growth cones (Filous et al., 2014). Some CSPGs, however, are permissive to axon growth (Busch et al., 2010; Miller and Hsieh-Wilson, 2015) and are also expressed by scar-forming astrocytes (Anderson et al., 2016). Interestingly, while the CSPG4 (also known as NG2) molecule is inhibitory (Dou and Levine, 1994; Tan et al., 2005; Filous et al., 2014), NG2^+^ oligodendrocyte progenitor cells can support axon growth (Yang et al., 2006). A substantial number of NG2 cells differentiate into scar-forming astrocytes after spinal cord contusion (Hackett et al., 2016, 2018), raising the possibility that NG2 cell-derived astrocytes at the lesion may have a higher capacity to facilitate axon regeneration (Hackett et al., 2018). Finally, after spinal cord injury, axons that grow into the lesion often associate with astrocytes that express laminin, an extracellular matrix molecule known to promote axon growth (Frisen et al., 1995; Ma et al., 2004; White et al., 2008, 2011). Fibronectin produced by astrocytes can also enhance axon regeneration in mature white matter (Tom et al., 2004). Injury-dependent re-expression of axon growth-favorable extracellular matrix proteins along with developmental markers in reactive astrocytes suggests a shift to a more immature cellular state that is conducive to axon regeneration (Silver et al., 1993; Filous et al., 2010).

### Clearance of cellular debris

Myelin debris persists chronically after CNS injury and is a source of myelin-associated inhibitors of axonal growth (Yiu and He, 2006; Geoffroy and Zheng, 2014). CNS myelin debris also stimulates inflammation to cause secondary tissue damage (Chen et al., 2000; Jeon et al., 2008; Tanaka et al., 2009; Sun et al., 2010; Allodi et al., 2012; Abiega et al., 2016; Zhou et al., 2021). Clearance of cellular debris including myelin-derived inhibitors after CNS injury, therefore, can facilitate axon regeneration. Microglia promote axon sprouting, through the proposed mechanism of phagocytosing myelin debris (Jiang et al., 2019). While microglia and monocyte-derived macrophages are the principal phagocytic cells in the injured CNS, reactive astrocytes can also become highly phagocytic by expressing phagocytic receptors and activation of phagocytic pathways early after spinal cord injury, traumatic brain injury, and brain ischemia (Morizawa et al., 2017; Konishi et al., 2020; Wang J. et al., 2020; Jiang et al., 2021; Zhou et al., 2021; Wan et al., 2022). Astrocytes can respond to neighboring damaged neurons through sensing and removal of cell debris in injured tissue, in a process that involves engulfment of debris in acidic endocytic vesicles directed toward lysosome for degradation (Wakida et al., 2018; Wang S. et al., 2020; Wan et al., 2022). Damaged neuron at the single-cell level is sufficient to trigger a “clean up” signal in nearby astrocytes. Astrocytes–microglia cooperation in cell corpse removal has been demonstrated to involve elimination of the cell body by microglia and axon-derived diffuse debris by astrocytes in the maintenance of brain homeostasis (Damisah et al., 2020). After ischemic injury, astrocyte-mediated phagocytosis temporally follows that by microglia and persists well into the subacute phase of injury (Morizawa et al., 2017). These observations suggest astrocytic contribution to cellular debris clearance that facilitates axon growth in the injured CNS.

### Bioenergetic support

Mitochondria supply ATP that is essential to neuronal survival and regeneration. Inter-cellular transfer of mitochondria contributes to provide metabolic requirements for axonal regeneration. Astrocytes are a source of functional extracellular mitochondria that support neuronal viability after ischemic stroke. Inhibition of extracellular mitochondria transfer through actin-dependent endocytosis results in failure of axonal growth (Hayakawa et al., 2016). Furthermore, astrocytes secret metabolites to support neuronal functions following CNS insults (Valenza et al., 2015; Chao et al., 2019). Lactate is one key metabolite secreted by astrocytes that acts as a signaling molecule to regulate neuronal functions that include synaptic plasticity and axonal integrity (Jha and Morrison, 2020). It was also demonstrated that lactate-treated astrocyte can induce axon outgrowth upon co**-**culture (Xu et al., 2020). Importantly, lactate has an essential role in axon regeneration after injury, and substitution of glucose by lactate supports axonal function and survival (Brown et al., 2012; Morrison et al., 2015). Local application of L-lactate, produced by glia including astrocytes under physiological conditions, enhances regeneration of corticospinal axons after spinal cord injury (Li et al., 2020).

## Astrocyte diversity: An astrocyte subtype that promotes axon growth?

The spectrum of astrocytic responses to injury—from isolation of tissue damage, inhibition or facilitation of axon growth, to immunomodulation among other functions—is highly contextual and needs not be conceived as contradictory. They depend on the injury type, phase of injury, location from the lesion, and cellular and molecular makeup of the local environment. Additionally, innate heterogeneity of astrocytes compounds the complexity of their injury response.

Regulation of astrocyte diversity in development by positional cues and neuronal activity has been comprehensively reviewed (Khakh and Deneen, 2019). Physiological adaptation of astrocytes, in form and function, to neural circuits has been well demonstrated (Chai et al., 2017). Evolution of astrocyte complexity was proposed to enable the integration and expansion of processing power of the human brain (Oberheim et al., 2006). scRNA-seq revealed regional astrocyte diversity, defined transcriptomically, in the healthy brain and spinal cord of mice and humans (Saunders et al., 2018; Hasel et al., 2021; Li et al., 2022; Sadick et al., 2022).

Following CNS insult, astrocytes from different brain regions mount region-specific transcriptional responses to the same stimulus, while also sharing common alterations in gene expression (Diaz-Castro et al., 2021). Interestingly, regional identity of astrocytes, as defined by gene expression signatures, is preserved even in a reactive state (Diaz-Castro et al., 2021). scRNA-seq confirmed that distinct astrocyte subtypes mount different transcriptional responses with temporal variation to the same CNS insult (Hasel et al., 2021; Li et al., 2022). Disease-specific interaction of transcriptional regulators together with engagement of core reactivity-promoting transcription factors in reactive astrocytes collectively contributes to differential gene expressions that determine disease-specific astrocytic phenotype (Burda et al., 2022).

Transcriptomic profiling has undoubtedly advanced understanding of astrocyte diversity. Molecular differences, however, do not always reflect functional alterations, knowledge of which remains rudimentary. For example, despite acute neuroinflammation inducing extensive gene expression changes in cortical astrocytes, modest effects on their electrophysiology, intracellular Ca^2+^ signaling, and gap junction coupling suggest retention of homeostatic functions (Diaz-Castro et al., 2021). Upregulation of genes associated with a neurotoxic astrocyte phenotype (Liddelow et al., 2017) did not correspond with neuronal loss or dysfunction (Diaz-Castro et al., 2021; Burda et al., 2022). Conversely, astrocyte dysfunction can manifest before or without upregulation of reactivity markers (Tong et al., 2014; Shandra et al., 2019). These findings highlight that while molecular signatures are one important identifier of astrocyte subtypes in disease, additional cellular properties and functional outcomes of reactive astrocytes are crucial to constructing a multidimensional understanding of astrocyte diversity in disease.

Astrocytes’ capacity to support axon growth, and therefore neural plasticity, in an injured CNS is a functional outcome important to CNS repair. While the extent to which this capacity is intrinsically determined remains to be explored, the local injury environment and the regenerative capacity of neurons likely influence astrocytes’ ability to promote axon growth. In acute focal injury, it is conceivable that the capacity of astrocytes to support regeneration of damaged axons at the injury site is low, whereas their capacity to support sprouting of spared axons away from the lesion is high. This is because the injury site contains tissue damage that results in a highly growth-inhibitory lesion core and CSPG accumulation (Davies et al., 1999; Lee et al., 2010; Zukor et al., 2013; Du et al., 2015; Anderson et al., 2016; Milich et al., 2021; Stern et al., 2021; Wahane et al., 2021). This regeneration-adverse environment, together with the inability of adult mammalian CNS neurons to regenerate damaged axons (Chen and Zheng, 2014), potentially creates a high barrier for astrocytes to effect axon growth at the primary lesion. In contrast, intact tissue remote from the injury site not only presents a growth-conducive environment, distal sprouting of intact axons remote from the lesion is also a spontaneous neuronal response to injury (Chen and Zheng, 2014). These conditions together conceivably lower the barrier for astrocytes to facilitate axon growth in spared tissue. Environment-dependent plasticity of reactive astrocytes has been demonstrated by conversion into scar-forming or naive phenotype following transplantation into injured or healthy spinal cords, respectively (Hara et al., 2017). The extent to which astrocytic capacity to support axon growth in an injured CNS is spatially determined, and how it can be harnessed therapeutically, would be of great interest for future investigation.

## Implications for future research

Reactive astrocytes, especially those located at the lesion border, have been widely regarded as inhibitory to regeneration in the adult mammalian CNS. Evidence is presented here to support a positive role of reactive astrocytes in axon growth in the injured mammalian CNS. Their beneficial effects on axon growth are consistent with the observations of exacerbated functional deficits following disruption of astrogliosis in rodent models (Faulkner et al., 2004; Okada et al., 2006; Herrmann et al., 2008). Recent gene expression data, by bulk or single-cell RNA sequencing, of reactive astrocytes collected at the lesion from acute to chronic phases of spinal cord injury will aid unbiased discovery of astrocyte-derived factors that can facilitate axon regeneration in the mature mammalian CNS (Anderson et al., 2016; Milich et al., 2021; Wahane et al., 2021; Wei et al., 2021; Brennan et al., 2022; Burda et al., 2022; Li et al., 2022). Given the long-recognized diversity of astrocytes, based on origin, location, injury type, and molecular signatures (Zhang and Barres, 2010; Khakh and Sofroniew, 2015), a future challenge will be to define context-dependent astrocyte functions, including their regulation of axon plasticity. Are there subtypes of reactive astrocytes with higher capacity to support axon growth after injury? Are pro-regenerative mechanisms differentially engaged by reactive astrocytes in a context-dependent manner to facilitate axon growth? While scar-forming astrocytes have the potential to facilitate axon growth, axon regeneration in the injured CNS is limited by the lack of neuron-intrinsic regenerative ability and an overwhelmingly growth-inhibitory lesion core. We propose that in contrast, stellate reactive astrocytes located in preserved tissue away from the lesion have greater potential to effect axon plasticity in the injured CNS and therefore a more attractive subtype of reactive astrocytes to target for neural repair. Molecular and functional profiling of astrocyte subtypes, and importantly, an integration of this information, will meaningfully advance understanding of astrocyte diversity and open avenues to effectively harness the beneficial functions of reactive astrocytes.

## Author contributions

All authors listed have made a substantial, direct, and intellectual contribution to the work, and approved it for publication.
